# Supplementary figures and images for: Allosteric coupling asymmetry mediates paradoxical activation of BRAF by type II inhibitors
Source: eLife. 2024 May 14;13:RP95481. doi: 10.7554/eLife.95481 (PMC11093583; doi:10.7554/eLife.95481)

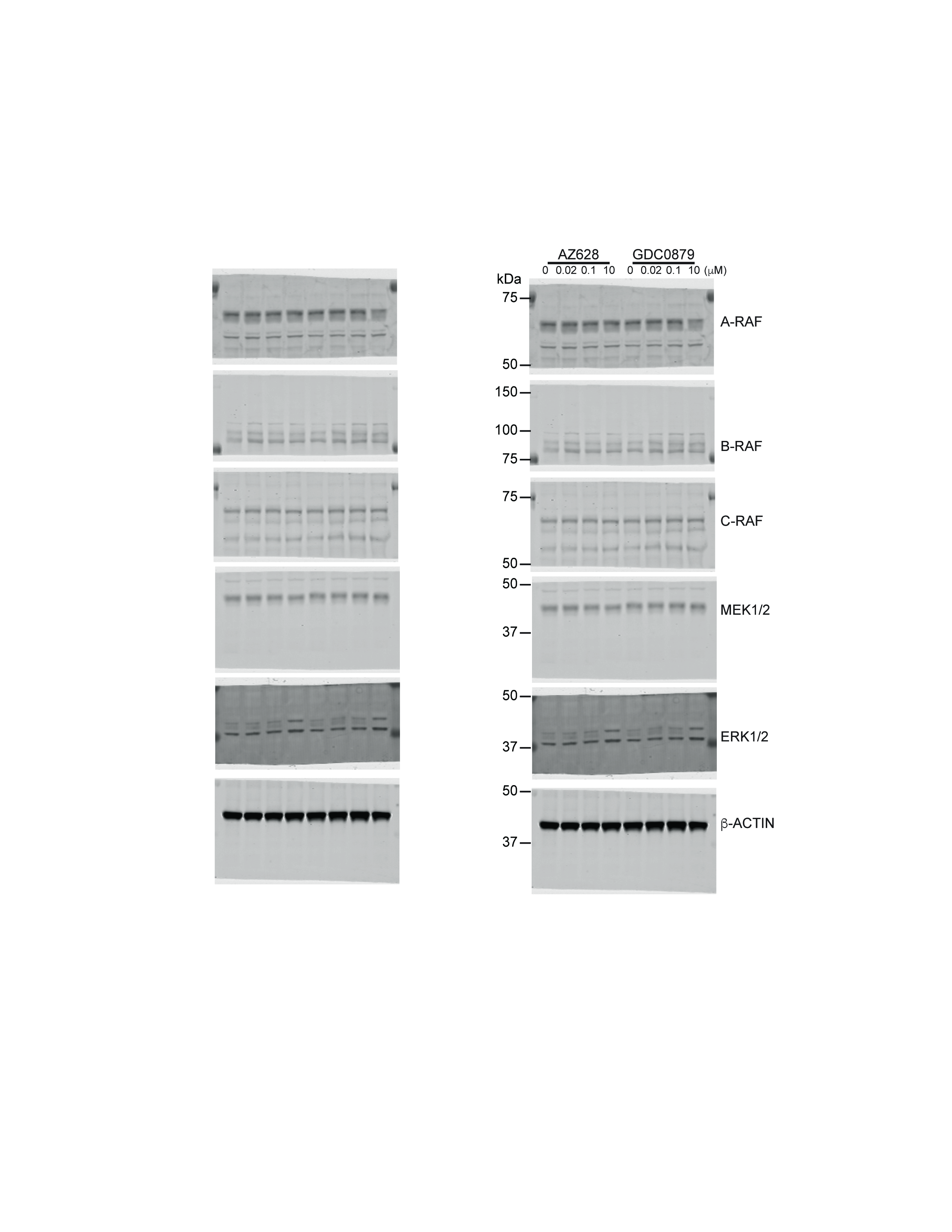

Supplement: Figure 3—figure supplement 3—source data 1. [file elife-95481-fig3-figsupp3-data1.zip › Figure 3_figure supplement 3 - source data 1.tif]

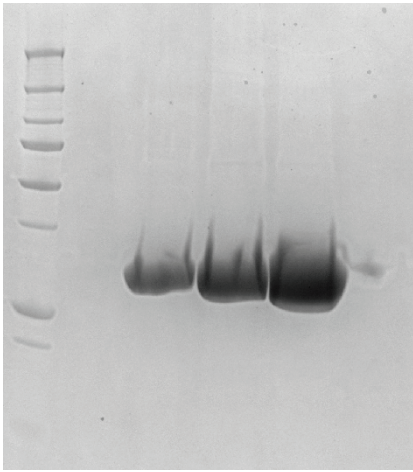

Supplement: Figure 4—figure supplement 2—source data 2. [file elife-95481-fig4-figsupp2-data2.zip › Figure 4_figure supplement 2 - source data 2.pdf]
