# Supplementary material for: Allosteric coupling asymmetry mediates paradoxical activation of BRAF by type II inhibitors
Source: eLife. 2024 May 14;13:RP95481. doi: 10.7554/eLife.95481 (PMC11093583; doi:10.7554/eLife.95481)
Supplement: Supplementary file 1. [file elife-95481-supp1.docx]

**Supplementary File I. Inhibitor sources and validation by mass spectrometry.**

| Inhibitor | source | monoisotopic mass [M+Na]+ | observed mass [M+Na]+ |
| --- | --- | --- | --- |
| vemurafenib | Selleck Chemicals | 512.06178 | 512.0394 |
| encorafenib | Selleck Chemicals | 562.14101 | 562.1418 |
| Dabrafenib | Selleck Chemicals | 542.0903 | 542.0917 |
| GDC0879 | Selleck Chemicals | 357.1322 | 357.1321 |
| SB590885 | TargetMol | 476.20571 | 476.204 |
| L779450 | Selleck Chemicals | 348.0898 | 348.0895 |
| Sorafenib Tosylate | Selleck Chemicals | 465.0936 | 465.093 |
| TAK632 | Selleck Chemicals | 577.0928 | 577.0924 |
| AZ628 | Selleck Chemicals | 474.19 | 474.1904 |
| LY3009120 | Selleck Chemicals | 447.2279 | 447.2273 |
| Ponatinib | TargetMol | 555.2091 | 555.2087 |
| ZM336372 | Selleck Chemicals | 412.1632 | 412.1631 |
| Belvarafenib | Selleck Chemicals | 501.0671 | 501.0675 |
| MLN2480 | Selleck Chemicals | 527.99947 | 528.0014 |
